# Crossbreeding Effect of Chalcogenation and Iodination on Benzene Additives Enables Optimized Morphology and 19.68% Efficiency of Organic Solar Cells

**DOI:** 10.1002/advs.202401405

**Published:** 2024-03-25

**Authors:** Tao Zhou, Wenwen Jin, Yinfeng Li, Xiaopeng Xu, Yuwei Duan, Ruipeng Li, Liyang Yu, Qiang Peng

**Affiliations:** ^1^ School of Chemical Engineering and State Key Laboratory of Polymer Materials Engineering Sichuan University Chengdu 610065 P. R. China; ^2^ College of Materials and Chemistry & Chemical Engineering Chengdu University of Technology Chengdu 610059 P. R. China; ^3^ National Synchrotron Light Source II Brookhaven National Lab Suffolk Upton NY 11973 USA

**Keywords:** chalcogenation and iodination, morphology optimization, noncovalent interaction, organic solar cells, volatile solid additive

## Abstract

Volatile solid additives have attracted increasing attention in optimizing the morphology and improving the performance of currently dominated non‐fullerene acceptor‐based organic solar cells (OSCs). However, the underlying principles governing the rational design of volatile solid additives remain elusive. Herein, a series of efficient volatile solid additives are successfully developed by the crossbreeding effect of chalcogenation and iodination for optimizing the morphology and improving the photovoltaic performances of OSCs. Five benzene derivatives of 1,4‐dimethoxybenzene (DOB), 1‐iodo‐4‐methoxybenzene (OIB), 1‐iodo‐4‐methylthiobenzene (SIB), 1,4‐dimethylthiobenzene (DSB) and 1,4‐diiodobenzene (DIB) are systematically studied, where the widely used DIB is used as the reference. The effect of chalcogenation and iodination on the overall property is comprehensively investigated, which indicates that the versatile functional groups provided various types of noncovalent interactions with the host materials for modulating the morphology. Among them, SIB with the combination of sulphuration and iodination enabled more appropriate interactions with the host blend, giving rise to a highly ordered molecular packing and more favorable morphology. As a result, the binary OSCs based on PM6:L8‐BO and PBTz‐F:L8‐BO as well as the ternary OSCs based on PBTz‐F:PM6:L8‐BO achieved impressive high PCEs of 18.87%, 18.81% and 19.68%, respectively, which are among the highest values for OSCs.

## Graphical abstract

Volatile benzene additives are developed by the crossbreeding effect of chalcogenation and iodination, which finely tune the molecular crystallinity and optimize the blend morphology, realizing a champion PCE of 19.68% in the ternary blend OSCs with SIB additive.

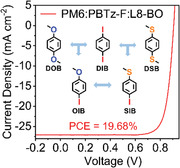

## 1 Introduction

Bulk heterojunction organic solar cells (BHJ‐OSCs) have witnessed rapid progress in the past years as a promising and versatile off‐grid energy supply candidate for applications in wearable, portable, and internet‐of‐things (IoT) integrated devices.^[^ ^1^, ^2^, ^3^, ^4^ ^]^ So far, the power conversion efficiencies (PCEs) over the 19% threshold have been successfully realized by continuous research efforts in material design and device engineering tactics.^[^ ^5^, ^6^, ^7^, ^8^, ^9^, ^10^ ^]^ It is well established that the photoactive layer plays a vital role in governing the photoelectric conversion process in the BHJ‐OSCs. Beyond the development of novel donor and acceptor materials with strong and complementary absorption as well as matched energy levels, delicate morphology control of the photoactive layer is equally important to achieve high performances of BHJ‐OSCs. This is because the exciton diffusion and dissociation, charge transportation, and recombination processes highly rely on the nanoscale morphology of the photoactive layer.^[^ ^11^, ^12^, ^13^, ^14^ ^]^ The most salient feature of an ideal morphology is recognized to be a bicontinuous interpenetrating network formed by the spontaneous phase separation of premixed donor and acceptor materials in the photoactive layer.^[^ ^15^, ^16^, ^17^, ^18^ ^]^ Nevertheless, we can hardly obtain such an ideal morphology in the as‐cast photoactive layer solely depending on the natural properties of donor and acceptor materials because the final morphology is not only determined by their inherent properties but also affected by different processing conditions.^[^ ^11^, ^17^ ^]^ Therefore, it is of great need to develop appropriate strategies to optimize the morphology according to the inherent properties of certain photoactive materials for maximizing the performances of BHJ‐OSCs.

The currently most efficient BHJ‐OSCs predominantly employ a polymer donor and a non‐fullerene acceptor (NFA) to form the photoactive layer. Unlike the formerly dominated fullerene acceptors, NFAs share similar π‐conjugated skeletons, and thus similar solubility to donor materials. This makes the solvent additives, which have ever been extensively used in fullerene‐based OSCs,^[^ ^19^ ^]^ confront challenges in optimizing the morphology of NFA‐based photoactive layers via the selective solubility mechanism.^[^ ^17^, ^20^ ^]^ Alternatively, employing volatile solid additives has been emerging as a potential method to optimize the morphology of currently prevailing NFA‐based OSCs.^[^ ^12^, ^21^, ^22^, ^23^, ^24^ ^]^ Volatile solid additives typically encompass a small aromatic core attached to various functional groups, making them have intrinsically high crystallinity and versatile noncovalent intermolecular interactions. Therefore, they can be functionalized as nucleation agents by offering crystal centers and introducing noncovalent bonds that crosslink the host materials.^[^ ^25^ ^]^ As a result, the crystallization of the photoactive materials is successfully modulated, thus enabling morphology optimization and photovoltaic performance enhancement.^[^ ^5^, ^22^, ^26^, ^27^, ^28^ ^]^ Moreover, a solid additive with high volatility is considered to be essential to make it readily removed from the photoactive layer by posttreatment so that it does not impact the operation stability of the devices like those low volatile solvent additives. As for the functional groups used in solid additives, halogen atoms (F, Cl, Br, and I) are most commonly investigated, and a number of efficient solid additives with halogen atoms have been developed by different research groups.^[^ ^17^, ^26^, ^28^, ^29^, ^30^, ^31^, ^32^, ^33^, ^34^, ^35^, ^36^, ^37^, ^38^, ^39^, ^40^, ^41^, ^42^, ^43^, ^44^ ^]^ These halogen atoms provide diverse weak‐bonding interactions with the photoactive materials for morphology optimization, and the volatilizable nature makes them easily removed to ensure morphological stability.^[^ ^28^, ^30^, ^34^ ^]^ In addition to halogen atoms, oxygen atoms have also been widely introduced to develop additives by some groups.^[^ ^12^, ^13^, ^14^, ^45^, ^46^, ^47^, ^48^, ^49^, ^50^ ^]^ In these additives, versatile forms of oxygen‐containing groups, such as alkoxy, hydroxyl, and carbonyl groups, provide various types of noncovalent interactions with the host materials for modulating the morphology. In contrast, although sulfur atom has been extensively introduced into the side chains of photoactive materials to improve the intermolecular interactions via noncovalent bonds such as S···S, S···F, S···O, S···N, etc., it is rarely used to develop volatile solid additives (regardless of that in the aromatic forms, such as in thiophene ring).^[^ ^12^, ^51^ ^]^ The application potential and working mechanism of sulfur atom‐containing additives remain to be disclosed. Moreover, the joint effect of chalcogenation and halogenation on the volatile solid additives also remains to be poorly understood.

In this work, we report a series of efficient volatile solid additives developed by the combination of chalcogenation and iodination on the benzene core for optimizing the morphology and improving the photovoltaic performances of OSCs. Five benzene derivatives were systematically investigated, including 1,4‐dimethoxybenzene (DOB), 1‐iodo‐4‐methoxybenzene (OIB), 1‐iodo‐4‐methylthiobenzene (SIB), 1,4‐dimethylthiobenzene (DSB) and 1,4‐diiodobenzene (DIB), where the widely used DIB was used as the reference. The different functional groups on these additives make them have different thermal properties and molecular interactions with the host photoactive materials. Compared to DIB, the other four additives possess much lower melting points (m.p.) below 100 °C, making them solid‐liquid convertible under the photoactive layer processing conditions. Thus, they can play multiple roles such as nucleation center at room temperature and plasticizer under thermal annealing (TA) processing in tailoring the morphology. Their lower boiling points (m.p.) and higher saturated vapor pressures (v.p.) also make them be removed easily upon TA treatment. Among them, SIB with a combination of sulfur and iodine atoms on SIB enabled more appropriate interactions with the host blend, thus finely optimizing the morphology. As a result, the binary OSCs based on PM6:L8‐BO and PBTz‐F:L8‐BO as well as the ternary OSCs based on PBTz‐F:PM6:L8‐BO achieved impressive high PCEs of 18.87%, 18.81% and 19.68%, respectively, which are among the highest performances for OSCs at present.

## 2 Results and Discussion

### 2.1 Thermal Properties Intermolecular Interactions

**Figure 1:**
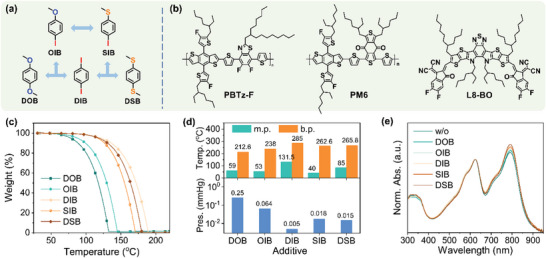
a) Design strategy and chemical structures of the additives. b) Chemical structures of the photoactive materials. c) TGA curves of the solid additives under a heating rate of 10 °C min^−1^. d) Column plots of the m.p., b.p., and saturated v.p. of the additives. e) UV–vis–NIR absorption of PM6:L8‐BO blend films without and with various additives.

The chemical structures of the additives and the photoactive materials are shown in **Figure** **1**a,b. All the additives have relatively high b.p. values over 200 °C (212.6, 238, 285, 262.6, and 265.8 °C for DOB, OIB, DIB, SIB, and DSB, respectively), making them able to retain in the active layer during film deposition at room temperature. Compared to the widely used DIB (a high m.p. of 131.5 °C), the other four molecules have relatively smaller m.p. (59, 53, 40, and 85 °C for DOB, OIB, SIB, and DSB, respectively) (Figure 1c), lower than the TA temperature commonly used in regulating the morphology (≈100 °C). Thus, they may play multiple roles such as nucleation centers at room temperature and plasticizers under TA processing in tailoring the BHJ nanoscale morphology.^[^ ^25^ ^]^ The saturated v.p. values are 0.25, 0.064, 0.005 0.018, and 0.015 mmHg for DOB, OIB, DIB, SIB, and DSB, respectively. The smallest b.p. and highest v.p. of DOB make it easily removed from the photoactive blend. On the contrary, DIB is more resistant to be removed than the other additives because of its largest b.p. and lowest saturated v.p. The removability of these additives was studied by thermogravimetric analysis (TGA), where the variation trend of weight loss agreed well with the variations of b.p. and saturated v.p. for these additives. Although they have high b.p. over 200 °C, all of them can be fully removed below 200 °C due to their volatile nature. Their differences in volatility might exert different impacts on the photoelectric properties of the host blend.

UV–vis–NIR absorption was first conducted to study the effect of these additives on the PM6:L8‐BO blend film (Figure 1d). Compared to the control film without additives, all the additives induced somewhat enhanced absorption of PM6:L8‐BO, especially at a longer wavelength, suggesting the more ordered molecular packing of the L8‐BO acceptor induced by these additives. Among them, DOB induced the minimum growth of absorption, while DSB triggered the maximum growth of absorption. The absorption strength of OIB‐modified film was in between DOB and DIB, and the absorption strength of SIB‐modified films was in between DSB and DIB. These results suggested their different intermolecular interaction abilities with the host blend, in which DOB had lower interactions, while DSB had stronger interactions with the host blend. The intermolecular interactions could be mediated by the crossbreeding effect of chalcogenation and iodination.

**Figure 2:**
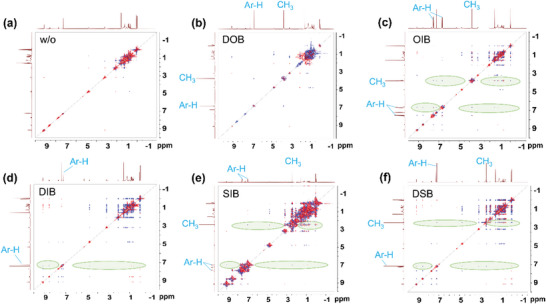
The 2D‐NMR NOESY spectra of a) neat L8‐BO and LB‐8O with b) DOB, c) OIB, d) DIB, e) SIB, and f) DSB additives. The marked Ar‐H and CH_3_ denoted the ^1^H resonance peaks from the benzene cores and the methyl groups of these additives, respectively. The crossover resonance signals between the additives and L8‐BO were marked with light green shadows.

2D ^1^H‐^1^H nuclear overhauser effect spectroscopy (NOESY) was used to verify the intermolecular interactions between the additives and the L8‐BO acceptor (**Figure** **2**).^[^ ^14^, ^36^ ^]^ No obvious crossover resonance signal was observed between L8‐BO and DOB, implying DOB might not be able to induce strong intermolecular interaction with L8‐BO. In contrast, adding the other four additives into the L8‐BO solution, multiple crossover resonance peaks were detected between not only the aromatic protons (belong to the end groups) but also the aliphatic protons (belong to the side chains on the central core) of L8‐BO and the H protons of these additives (Figure 2c–f, highlighted by light green shadows). The results suggested that these additives (except for DOB) could induce noteworthy intermolecular interactions with both the central core and the terminals of L8‐BO. The different functional groups could induce variations in the molecular interaction strength, offering opportunities to finely tune the morphology of the corresponding blend films.

### 2.2 Photovoltaic Properties

**Figure 3:**
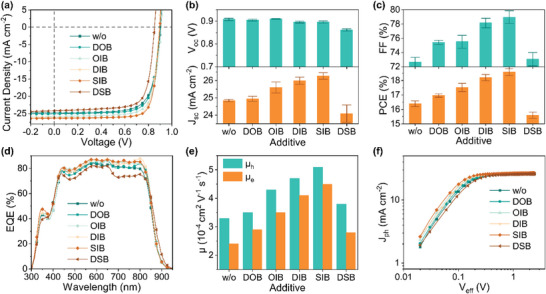
a) *J–V* curves, b) Column plots of *V*_oc_ and *J*_sc_ distributions, c) Column plots of FF and PCE distributions, and d) EQE spectra of the different additives modified OSCs. e) Column plots of charge carrier mobilities for the PM6:L8‐BO films modified with various additives. f) *J*_ph_ versus *V*_eff_ plots of the different additives modified OSCs.

OSCs based on a device structure of glass/ITO/PEDOT:PSS/PM6:L8‐BO/PNDIT‐F3N/Ag were fabricated to study the efficiency improvement ability of these additives (see Supporting Information for fabrication details). Without any additive, the control devices produced a high *V*_oc_ of 0.907 V but a relatively low *J*_sc_ of 24.77 mA cm^−2^ and FF of 73.36%, limiting the PCE to 16.49% (**Figure** **3** and **Table** **1**). Introducing the widely used DIB as the additive, a comparable high *V*_oc_ of 0.893 V was obtained, but the *J*_sc_ and FF were largely improved to 26.17 mA cm^−2^ and 79.43%. As a result, the PCE was significantly improved to 18.57%. In contrast, only slightly improved PCE of 17.05% was obtained for the DOB additive modified devices due to the mildly increased *J*_sc_ of 25.05 mA cm^−2^ and FF 75.60% than the control devices. If employing DSB as the additive, the almost comparable *J*_sc_ of 24.60 mA cm^−2^ and FF 73.90% were obtained compared to the control devices, but the *V*_oc_ was largely reduced to 0.858 V, thus reducing the PCE to 15.59%. Fortunately, by crossbreeding of chalcogenation and iodination in the additives, the device performances were more effectively improved. Specifically, replacing DOB with OIB additive, the devices achieved higher *V*_oc_ of 0.910 V, *J*_sc_ of 25.86 mA cm^−2^ and FF of 75.78%, giving rise to the elevated PCE of 17.83%. Moreover, if introducing the SIB additive, the devices realized further increased *J*_sc_ of 26.26 mA cm^−2^ and FF of 79.92%, thus delivering a champion PCE of 18.87% with a decent *V*_oc_ of 0.898 V. The effect of these additives on the photovoltaic performances was further studied by the external quantum efficiency (EQE) spectra (Figure 3d). The addition of these additives, except for DSB, notably increased the EQE response of the related OSCs throughout the absorption range, confirming their improved *J*_sc_ values. The calculated current density (*J*_EQE_) was 24.27 mA cm^−2^ for the control devices without additives, which was increased to 24.74, 25.12, 25.39, and 25.46 mA cm^−2^ for the devices modified with DOB, OIB, DIB, and SIB, respectively. In contrast, the DSB‐based devices exhibited obviously reduced EQE response, especially in longer wavelengths, producing a lower *J*_EQE_ of 23.87 mA cm^−2^. The results suggest rationally introducing chalcogen and halogen atoms on the benzene core is crucially important to developing efficient additives.

**Table 1:** Photovoltaic parameters of OSCs containing PM6:L8‐BO layer modulated with various additives.

Additives	*V*_oc_ [V]	*J*_sc_ [mA cm^−2^]	*J*_EQE_ ^b)^ [mA cm^−2^]	FF [%]	PCE [%]
w/o	0.907 × 0.908 ± 0.005^a)^	24.77 × 24.84 ± 0.07	24.27	73.36 × 72.65 ± 0.69	16.49 × 16.39 ± 0.20
DOB	0.900 × 0.905 ± 0.004	25.05 × 24.94 ± 0.15	24.74	75.60 × 75.41 ± 0.30	17.05 × 16.96 ± 0.12
OIB	0.910 × 0.910 ± 0.002	25.86 × 25.60 ± 0.32	25.12	75.78 × 75.52 ± 0.90	17.83 × 17.52 ± 0.28
DIB	0.893 × 0.896 ± 0.004	26.17 × 25.99 ± 0.20	25.39	79.43 × 78.15 ± 0.67	18.57 × 18.20 ± 0.22
SIB	0.898 × 0.896 ± 0.005	26.30 × 26.27 ± 0.19	25.46	79.92 × 78.96 ± 0.91	18.87 × 18.58 ± 0.24
DSB	0.858 × 0.861 ± 0.005	24.60 × 24.08 ± 0.50	23.87	73.90 × 73.08±0.94	15.59 × 15.15 ± 0.22

^a)^ The averaged values with standard deviations were calculated from 10 individual devices;

^b)^ The integrated current densities were calculated from the EQE curves.

The effect of the additive on device stability was investigated by maximum power point tracking under continuous white light‐emitting diode (LED) illumination (Figure S1, Supporting Information). After 250 h of continuous illumination, the control devices without additives and the devices with SIB and DIB additives retained ≈86%, 87%, and 90% of the original PCEs, respectively. Therefore, the SIB additive could have a positive effect on improving not only the device's performance but also the device's stability.

To study the chalcogenation and iodination effects of these additives on the charge transport properties of PM6:L8‐BO thin films, space charge limited current (SCLC) measurements were conducted (Figure S2, Supporting Information). As shown in Figure 2e and Table S1 (Supporting Information), the control film without additive showed a hole mobility (*µ*_h_) of 3.3 × 10^−4^ cm^2^ V^−1^ s^−1^ and an electron mobility (*µ*_e_) of 2.4 × 10^−4^ cm^2^ V^−1^ s^−1^. After adding the additives, the *µ*_h_/*µ*_e_ values were increased to 3.5 × 10^−4^/2.9 × 10^−4^ cm^2^ V^−1^ s^−1^ for DOB, 4.3 × 10^−4^/3.5 × 10^−4^ cm^2^ V^−1^ s^−1^ for OIB, 4.7 × 10^−4^/4.1 × 10^−4^ cm^2^ V^−1^ s^−1^ for DIB, 4.7 × 10^−4^/4.1 × 10^−4^ cm^2^ V^−1^ s^−1^ for SIB, and 3.8 × 10^−4^/2.8 × 10^−4^ cm^2^ V^−1^ s^−1^ for DSB modified devices, respectively. Among them, SIB‐modified devices obtained the highest *µ*_h_ and *µ*_e_ values, suggesting the more efficient charge transport for suppressing the charge recombination probability and improving the photovoltaic performances.

To further study the chalcogenation and iodination effects of these additives on the device performances, photocurrent (*J*_ph_) versus effective voltage (*V*_eff_) were plotted, and charge dissociation efficiency (*P*_diss_) under short‐circuit condition and collection efficiency (*P*_coll_) under the maximum power point (MPP) were calculated (Figure 2f). The *P*_diss_/*P*_coll_ were determined to be 96.9%/88.7% for the control devices, which were gradually increased to 97.4%/90.6%, 98.1%/91.2%, 98.5%/93.3%, 98.6%/93.6% for the DOB, OIB, DIB, and SIB modified devices, respectively. The results confirmed the improved charge dissociation and collection in the devices, thus enabling higher device performances. In contrast, the DSB‐based devices exhibited a reduced *P*_diss_/*P*_coll_ of 96.1%/88.2%, suggesting the lowered charge dissociation and collection in devices, thus leading to inferior photovoltaic performances.

### 2.3 Morphology Studies

**Figure 4:**
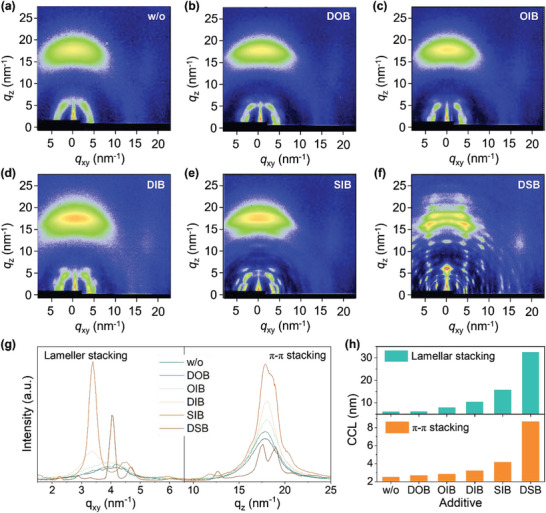
a–f) GIWAXS patterns of PM6:L8‐BO thin films modified with different additives. g) In‐plane and out‐of‐plane line cuts from (a–f). h) CCL plots of diffraction peaks for PM6:L8‐BO thin films with various additives.

Grazing incidence wide‐angle X‐ray scattering (GIWAXS) experiments were conducted to study the chalcogenation and iodination effects of these additives on the molecular packing behaviors of the photoactive materials (**Figure** **4**; Figure S3, Supporting Information). Without introducing any additive, the control L8‐BO thin film exhibited a well‐defined (11‐1) diffraction peak around *q*_xy_ = 4.1 nm^−1^ in the in‐plane direction, and (010) diffraction peak around *q*_xy_ = 17.8 nm^−1^ and out‐of‐plane direction, which were assigned to the lamellar and π–π stackings, respectively (Table S2, Supporting Information). The crystal coherence lengths (CCLs) for the lamellar and π–π stackings were estimated to be 6.07 and 2.50 nm, respectively. The addition of DOB and OIB showed mildly impact on the molecular packing of L8‐BO, as quantified by slightly increased CCLs for the lamellar/π–π stackings (3.16/2.68 nm for DOB and 7.89/2.81 nm for OIB modified films). In contrast, the other three additives exerted a noteworthy intensified molecular packing of L8‐BO. After being modified by DIB, the *q* vector for π–π stacking of L8‐BO was increased to 18.0 nm^−1^ with a larger CCL of 3.20 nm, showing the more compact and ordered π–π stacking. In addition to the (11‐1) peak around *q*_xy_ = 4.30 nm^−1^ (CCL = 10.4 nm), one more peak was observed at a lower q vector (*q*_xy_ = 3.36 nm^−1^) in the DIB modified film, which might come from the (110) diffraction, suggesting the multiple packing modes of L8‐BO.^[^ ^52^ ^]^ Interestingly, a much higher (110) diffraction peak was observed in the SIB‐modified film, along with largely enhanced (11‐1) diffraction (CCL = 15.7 nm) and (010) diffraction (CCL = 4.14 nm), and the (11‐1) diffraction shifted to a higher q vector (*q*_xy_ = 4.48 nm^−1^). The results demonstrated the further intensified molecular packing of L8‐BO via interaction with SIB. While adding DSB as the additive, many diffraction peaks could be observed throughout the GWAXS image, showing its highest crystallinity with more ordered crystals formed in the film. The results indicated a stronger intermolecular interaction between DSB and L8‐BO. The impact of these additives on the PM6 donor was relatively lower than that on the L8‐BO acceptor (Figure S2, Supporting Information) but held the same trend observed in the latter. Especially, compared with other conditions, PM6 film modified with DSB exhibited one more semicircle diffraction around *q* = 8.5 nm^−1^, suggesting the different packing modes of PM6 induced by DSB. The variations in molecular crystallinity and packing modes induced by these additives could provide sufficient driving force for morphology modulation (vide infra).

**Figure 5:**
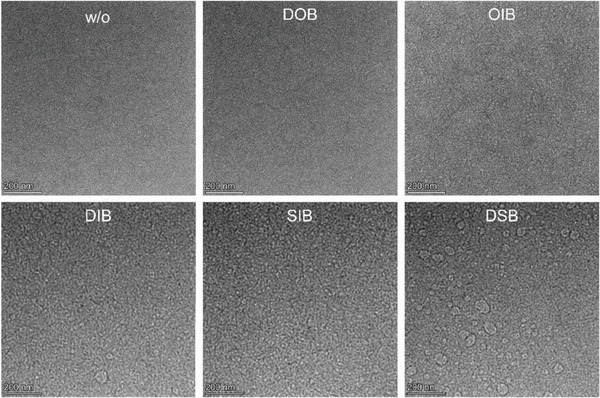
a–f) TEM images of the PM6:L8‐BO thin films modified with and without different additives.

The effect of additives on the blend morphology was further investigated by transmission electron microscopy (TEM) measurements (**Figure** **5**). A relatively low‐contrast TEM image was obtained for the control PM6:L8‐BO thin film without additive modification, suggesting its relatively weak phase separation, which was improved by the commonly used DIB additive as expected. While compared with the control PM6:L8‐BO thin film, using DOB as the additive, the TEM image displayed no obvious change, indicating DOB was not powerful enough as DIB to force the crystallization of donor and acceptor for improving the phase separation. On the contrary, the modified film generated a high‐contrast TEM image with varying sizes of large domains distributed throughout the image, indicating the stronger phase separation over aggregation of the photoactive components in the thin film. The results were consistent with the 2D‐NOSY and GIWAXS results, where DOB had less obvious interaction with the photoactive materials while DSB had very strong interaction with the photoactive materials, resulting in the differences in crystallinity and phase separation of the PM6:L8‐BO film. Moreover, the interaction strength and molecular crystallinity were subtly mediated by the cross‐dressing effects of chalcogenation and iodination. Specifically, the weak phase separation of DOB‐based film was successfully improved by cross‐dressing of oxygen and iodine atoms (OIB), and the over‐segregation of DSB‐based film was satisfactorily reduced by the cross‐dressing of sulfur and iodine atoms (SIB). Among them, the SIB additive not only improved the crystallinity to a relatively high level but also finely optimized the phase separation with suitable domain sizes, thus benefitting the efficient exciton dissociation and charge transport. The results explained well the differences in the device performances and highlighted the importance of rational design of additives with appropriate functional groups.

### 2.4 Charge Transfer Dynamics

**Figure 6:**
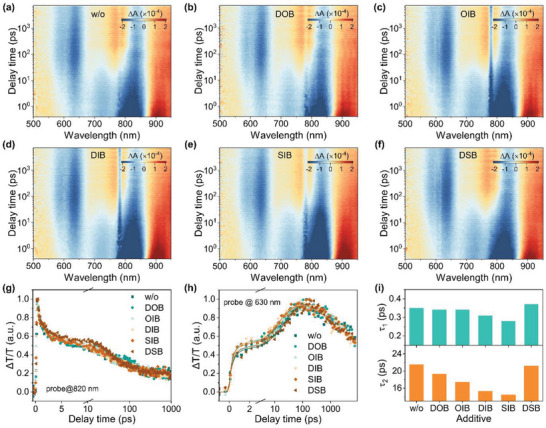
a–f) fs‐TA spectra of the PM6:L8‐BO films modified with various additives. g) TA kinetics of the GSB signals at 820 nm of the above PM6:L8‐BO films. h) TA kinetics of the GSB signals at 630 nm of the above PM6:L8‐BO films. i) Column plots of the τ_1_ and τ_2_ estimated from fitting the TA kinetics.

Femtosecond transient absorption spectroscopy (fs‐TAS) experiments were conducted to get insight into the chalcogenation and iodination effects of these additives on the charge transfer dynamics of the photoactive layer (**Figure** **6**). A 780 nm laser light was used to selectively excite the L8‐BO acceptor, which produced excited‐state absorption (ESA) and ground‐state bleach (GSB) decay traces around 820 and 920 nm, respectively.^[^ ^17^ ^]^ In addition, the signals raised first and then decayed ≈550–650 nm was assigned to the GSB of PM6 donor, suggesting the hole‐transfer process from L8‐BO to PM6. After adding additives, faster GSB decay rates of L8‐BO were observed, resulting in faster GSB rises of PM6 donors (Figure 6 g,h). By fitting the rising kinetics of PM6 GSB signals at 630 nm via a bi‐exponential function, the fast component (τ_1_) and slow component (τ_2_) could be obtained, where the former denoted the kinetics of the exciton dissociation at the D/A interfaces, and the latter represented the kinetics of exciton diffusion in crystalline domains. All these photoactive layers had a quite small τ_1_ below 0.4 ps (0.35 ps for the control blend without additive, 0.34, 0.34, 0.31, 0.28, and 0.37 ps for the DOB, OIB, DIB, SIB, and DSB modified blends, respectively) (Figure 6i), suggesting their fast hole transfer at the PM6/L8‐BO interfaces. In contrast, the rate‐limiting step was the exciton diffusion process. The control PM6:L8‐BO blend without additive had a relatively large τ_2_ of 21.5 ps. By adding the additives, τ_2_ was reduced to 21.5, 19.3, 15.3, 14.4, and 21.2 ps for DOB, OIB, DIB, SIB, and DSB‐modified photoactive layers, respectively. Among them, DSB film led to a slightly larger τ_1_ and smaller τ_2_ compared to the control photoactive layer. The reduced τ_2_ could be due to the improved crystallinity of L8‐BO, while the increased τ_1_ might be caused by the aggregation and multiphased of L8‐BO induced by adding DSB, resulting in reduced D/A interfaces and morphological traps for impeding the hole transfer from L8‐BO to PM6. In contrast, the addition of SIB realized smaller τ_1_ and τ_2_, suggesting more efficient charge transfer due to the enhanced D/A interaction in the mixed phase and the enhanced crystallinity. Therefore, higher photovoltaic performances were realized in SIB‐modified OSCs.

### 2.5 Applications in All‐PSCs and Ternary OSCs

All‐polymer solar cells (all‐PSCs) have received increasing attention in the past years due to their additional merits of better film‐forming properties, morphological robustness, mechanical durability, and operational stability than the small molecule acceptor‐based OSCs.^[^ ^53^ ^]^ To investigate the application potential of SIB additive in all‐PSCs, the related devices based on PBBTz‐Cl:PY‐IT were fabricated and evaluated (Figure S4 and Table S3, Supporting Information), where the wide bandgap polymer donor of PBBTz‐Cl was previously developed by our group.^[^ ^53^ ^]^ The control devices without any additive obtained a moderate PCE of 15.63%, which was significantly improved to 17.47% after adding 50 wt.% of SIB additive in the active layer. These results demonstrated the great application potential of SIB in optimizing the morphology and photovoltaic performances of all PSCs.

**Figure 7:**
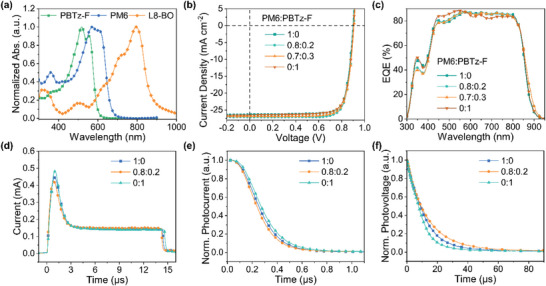
a) Normalized absorption of the photoactive materials. b) *J–V* curves of the binary and ternary OSCs optimized by SIB additive. c) EQE spectra of the binary and ternary OSCs optimized by SIB additive. d) Photo‐CELIV, e) Normalized TPC curves, and f) Normalized TPV curves of the binary and ternary blend OSCs optimized by SIB additive.

Ternary OSCs show great potential in developing efficient OSCs by taking advantage of the vast existing pool of organic semiconductors with various bandgaps and aggregation properties. Thus, ternary OSCs offer an opportunity to overcome the absorption limitation and finely tune the blend morphology for further improving the performance. Herin, one efficient polymer of PBTz‐F (Figure 1b)^[^ ^54^ ^]^ was introduced as a second donor to study the application potential of SIB additive in the tenantry OSCs. Compared with PM6, PBTz‐F has strong absorption within shorter wavelength regions, making it have the potential to improve light harvesting in the ternary devices (**Figure** **7**a). To our excitement, employing SIB as the additive in the PBTz‐F:L8‐BO‐based devices, an impressive PCE of 18.81% was realized, with a high *V*_oc_ of 0.905 V, *J*_sc_ of 26.57 mA cm^−2^ and FF of 78.23% (Figure 7b; Table S4, Supporting Information). When adding 20wt.% of PBTz‐F (relative to the total donor weight) into the PM6:L8‐BO host blend, the enhanced *J*_sc_ of 27.10 mA cm^−2^ and FF of 80.06% contributed to the significantly improved PCE of 19.68%, which is among the highest performances of OSCs at present. Adding 30wt.% of PBTz‐F, the related devices could still realize a high PCE of 19.37%, showing the good miscibility of the two donors. The improved device performance in the ternary OSCs was also confirmed by the EQE measurements (Figure 7c), where the optimal ternary blend devices achieved a higher JEQE of 25.90 mA cm^−2^ than the other devices.

To get insight into the charge transport properties of ternary blend devices, photogenerated charge extraction by linearly increasing voltage (photo‐CELIV) was also carried out (Figure 7d). The extracted charge carrier mobilities (*µ*_celiv_) were estimated to be 1.56 × 10^−4^ cm^2^ V^−1^ s^−1^ for PM6:L8‐BO, and 1.42 × 10^−4^, cm^2^ V^−1^ s^−1^ for PBTz‐F:L8‐BO binary blend devices, respectively, which were increased to 2.01 × 10^−4^ cm^2^ V^−1^ s^−1^ for the optimal ternary blend devices. The charge carrier extraction property was studied by transient photocurrent (TPC) experiments (Figure 7e). The charge extraction time was calculated to be 0.17 and 0.19 µs PM6:L8‐BO and PBTz‐F:L8‐BO binary blend devices, respectively, which was decreased to 0.16 µs for the optimal ternary blend devices. In addition, the charge carrier lifetime estimated by transient photovoltage was 12.0 and 9.8 µs PM6:L8‐BO and PBTz‐F:L8‐BO binary blend devices, respectively, which was increased to 13.0 µs for the optimal ternary blend devices. The improved charge carrier mobilities and lifetime as well as decreased charge extraction time of the ternary blend devices account for the enhanced device performance. These results demonstrated the great application potential of SIB in optimizing the morphology and photovoltaic performances of ternary OSCs.

## 3 Conclusion

In summary, a series of efficient volatile solid additives were successfully developed by the crossbreeding effect of chalcogenation and iodination for optimizing the morphology and improving the photovoltaic performances of OSCs. Five benzene derivatives were systemically studied, including DOB, OIB, SIB, DSB, and DIB, where the widely used DIB was used as the reference. Compared to DIB, the other four additives possess much lower m.p. making them solid‐liquid convertible during active layer deposition. Thus, they could act as nucleation centers at room temperature and plasticizers under TA treatment to tailor the morphology. Their lower m.p. and higher v.p. also make them easily removed upon TA treatment. The results indicated that the methoxy groups on the DOB additive exerted almost no interaction with the host materials, while the methylthio groups on the DSB additive induced the strongest interaction with the host materials. Their interaction properties could be mediated by just replacing one methoxy or methylthio group with the iodine atom. The crossbreeding of chalcogenation and iodination enabled the finely tuned intermolecular interactions for morphology optimization. Among them, SIB with the combination of sulphuration and iodination induced more appropriate interactions with the host blend, giving rise to a highly ordered molecular packing and more favorable morphology. As a result, binary OSCs based on PM6:L8‐BO and PBTz‐F:L8‐BO as well as ternary OSCs based on PBTz‐F:PM6:L8‐BO achieved impressive high PCEs of 18.87%, 18.81%, and 19.68%, respectively, which are among the highest values for OSCs so far.

## Conflict of Interest

The authors declare no conflict of interest.

## Supporting information

Supporting Information

## Data Availability

Research data are not shared.
