# Supplementary material for: Crossbreeding Effect of Chalcogenation and Iodination on Benzene Additives Enables Optimized Morphology and 19.68% Efficiency of Organic Solar Cells
Source: Adv Sci (Weinh). 2024 Mar 25;11(23):2401405. doi: 10.1002/advs.202401405 (PMC11186042; doi:10.1002/advs.202401405)
Supplement: Supplementary file 1 — Supporting Information [file ADVS-11-2401405-s001.pdf]

ADVANCED SCIENCE
Open Access

Supporting Information

for *Adv. Sci.*, DOI 10.1002/advs.202401405

Crossbreeding Effect of Chalcogenation and Iodination on Benzene Additives Enables Optimized Morphology and 19.68% Efficiency of Organic Solar Cells

Tao Zhou, Wenwen Jin, Yinfeng Li, Xiaopeng Xu, Yuwei Duan, Ruipeng Li, Liyang Yu and Qiang Peng**

Supporting Information

Crossbreeding Effect of Chalcogenation and Iodination on Benzene Additives Enables Optimized Morphology and 19.68% Efficiency of Organic Solar Cells

Tao Zhou, Wenwen Jin, Yinfeng Li, Xiaopeng Xu, Yuwei Duan, Ruipeng Li, Liyang Yu and Qiang Peng**

T. Zhou, W. Jing, Y. Li, Dr. P. Xu, Dr. L. Yu, Prof. Q. Peng
School of Chemical Engineering and State Key Laboratory of Polymer Materials Engineering, Sichuan University, Chengdu 610065, P. R. China.
Email: xpxu@scu.edu.cn; qiangpeng@scu.edu.cn

Dr. Y. Duan, Prof. Q. Peng
College of Materials and Chemistry & Chemical Engineering, Chengdu University of Technology, Chengdu 610059, P. R. China.

Dr. R. Li
National Synchrotron Light Source II Brookhaven National Lab, Suffolk, Upton, NY 11973, USA

1. Materials and Methods

Materials: PM6 was purchased from Solarmer Materials Inc. L8-BO was purchased from Vizuchem Inc. PBTz-F was prepared by our group.[1] All the other chemicals were purchased from Aladdin, Adamas, Sigma-Aldrich and Alfa Asear Chemical Co., and used without further purification.

Methods: Thermogravimetric analysis (TGA) was conducted on a TA Instrument Model SDT Q500 at a heating rate of 10 ^{o}C min^{-1} and under a N_2 flow rate of 90 mL min^{-1}. UV-vis-NIR spectra were obtained on a Shimadzu UV3600 spectrophotometer. ^{1}H NMR was record from

a JNM-ECZ400S nuclear magnetic resonance spectrometer using $CDCl_3$ as the solvent. GIWAXS measurements were performed at Complex Materials Scattering (CMS) beamline of the National Synchrotron Light Source II (NSLS-II), Brookhaven National Lab. AFM images were obtained by using a Bruker Inova atomic microscope in tapping mode. TEM images were obtained by using a Thermo Scientific Talos F200S G2 transmission electron microscope. Femtosecond transient absorption (fs-TA) spectroscopy experiments were performed using a home-built system with a Ti: sapphire regenerative amplified laser system (Coherent Legend Elite). The probe beam was generated by focusing part of the fundamental femtosecond laser beam onto a 3-mm-thick sapphire plate or 4 mm-thick Yttrium aluminum garnet plate for visible (vis) and near-IR (NIR) spectral windows, respectively. 780 nm laser was used to selectively excite NFAs. TA results in this work were presented in the unit of ΔOD, negative features could reflect ground-state bleaching (GSB) or stimulated emission (SE), a positive signal was an excited-state absorption (ESA). During TA measurements, the samples were kept in nitrogen to avoid photodegradation. The pump fluence was kept at <5 μJ cm^{-2} to minimize the exciton−exciton annihilation effect.

2. Device Fabrication and Measurements

Device fabrication: The patterned indium tin oxide (ITO, sheet resistance = 15 Ω $square^{-1}$) glass substrates were sequentially ultrasonicated with detergent, deionized water, acetone and isopropanol. Then, the ITO glasses were treated with UV-ozone for 30 min. Poly(3,4-ethylenedioxythiophene):polystyrene sulfonate (PEDOT:PSS) (Bay PVP. Al 4083, Bayer AG) was filtrated through a 0.45 μm nylon filter and then spin-coated on the cleaned ITO substrates at 5000 rpm for 60 s to form a thin layer (35 nm). After that, the substrates were baked at 150 oC on a hot plate for 10 min. The PM6:L8-BO solutions (1:1.2 by weight, 16.5 mg/mL in chloroform) or PBTz-F:PM6:L8-BO solutions (x:(1-x):1.2 by weight, 16.5 mg/mL in chloroform) without and with different additives were stirred at room temperature for 2

hours before used. The above solutions were spin-coated on the ITO/PEDOT:PSS substrates at a speed of 3000 rpm for 30 s to form a ~100 nm thickness of the photoactive layer. Then, the substrates were baked at 100 ^{o}C for 10 min. PNDIT-F3N solution (0.5 mg/mL in methanol with 5 v% of acetic acid) was spin-coated on the top of the active layer to form a thin cathode interlayer. Finally, argentum electrode (Ag, 100 nm) was deposited under high vacuum (~10^{-5} Pa) in an evaporation chamber. For the hole-only devices, after deposition of the photoactive layer on ITO/PEDOT:PSS substrates, molybdenum oxide (10 nm) and Ag electrode (100 nm) were was deposited under high vacuum (~10^{-5} Pa) in an evaporation chamber. For the electron-only devices, diethylzinc solution (2 M in toluene diluted by tetrahydrofuran) was spin-coated on the ITO substrates under dry air followed by baked at 200 ^{o}C for 30 min, the other fabrication processes were identical to the OSC devices.

Device measurements: The *I-V* characterization was performed on a computer-controlled Keithley 2400 Source under AM1.5G (100 mW cm^{-2}) using a solar simulator (XES-70S1, SAN-EI), which was calibrated by a standard Si solar cell (AK-200, Konica Minolta, Inc.). The EQE values were measured with an EQ-R solar quantum efficiency test system (Enlitech Co., Ltd., Taiwan, China). All fabrication and characterization processes, except for the HTLs preparation and EQE measurements, were conducted in a high purity argon filled glove box.

3. Supplementary Figures

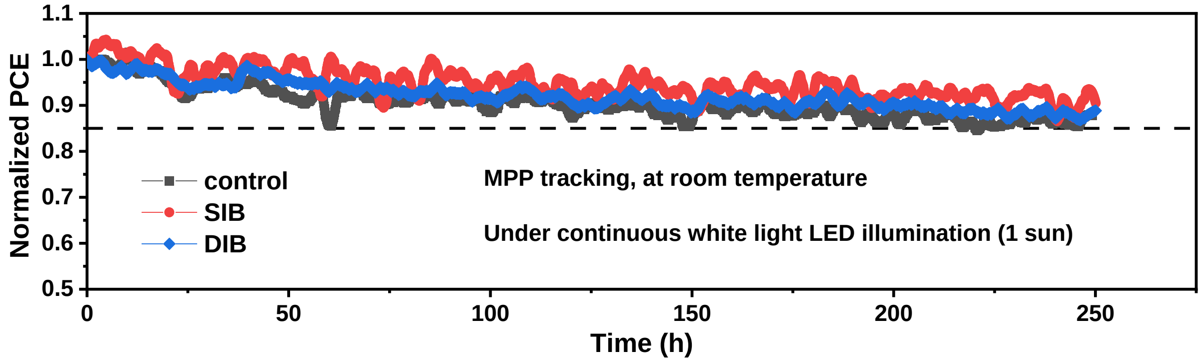

Figure S1. Normalized PCEs of the devices without additive and with SIB, DIB additive, respectively, under continuous white light LED illumination.

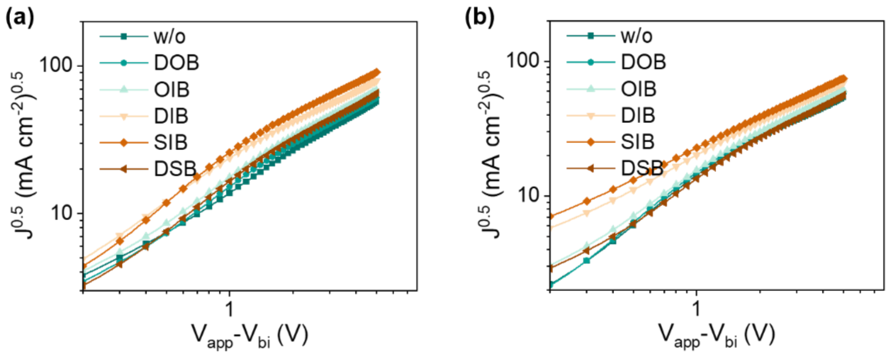

Figure S2. $J^{0.5}$-V curves of the (a) hole-only and (b) electron-only devices.

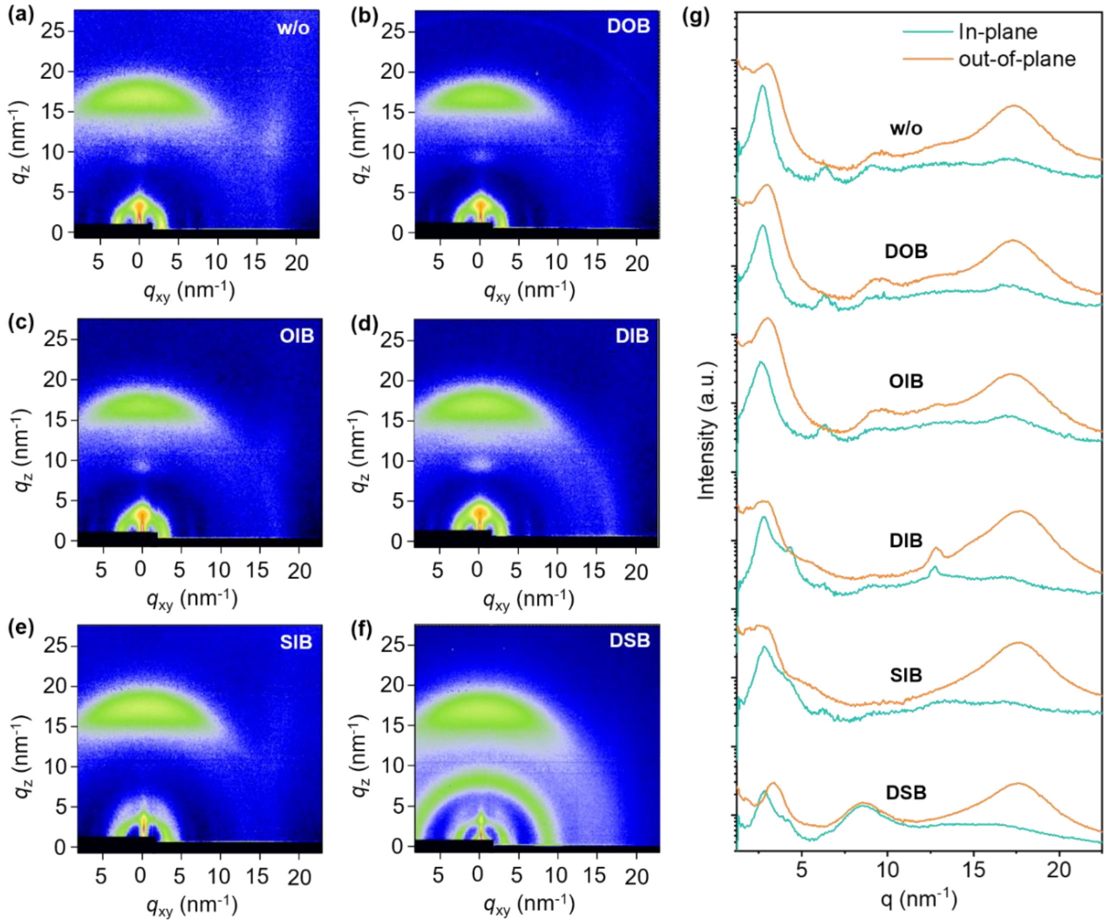

Figure S3. a-f) GIWAXS patterns of PM6 films modified with various additives. g) The corresponding in-plane and out-of-plane line-cuts from (a-f).

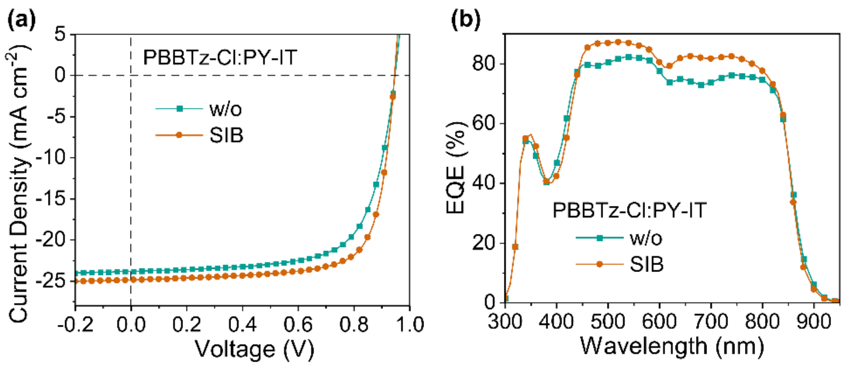

Figure S4. a) *J-V* curves of the PBBTz-Cl:PY-IT based all-PSCs without and with SIB additive modification. c) EQE spectra of the PBBTz-Cl:PY-IT based all-PSCs without and with SIB additive modification.

3. Supplementary Tables

Table S1. Charge carrier mobilities of the PM6:L8-BO blends with various additives.

Additive	μ_h [cm^2 V^{-1} s^{-1}]	μ_e [cm^2 V^{-1} s^{-1}]
w/o	3.3×10^{-4}	2.4×10^{-4}
DOB	3.5×10^{-4}	2.9×10^{-4}
OIB	4.3×10^{-4}	3.5×10^{-4}
DIB	4.7×10^{-4}	4.1×10^{-4}
SIB	5.1×10^{-4}	4.5×10^{-4}
DSB	3.8×10^{-4}	2.8×10^{-5}

Table S2. Summarized parameters for the ordered structures of L8-BO with various additives

Additive	Lamellar stacking			π-π stacking		
	q/d [nm^{-1}]/[nm]	FWHM [nm^{-1}]	CCL [nm]	q/d [nm^{-1}]/[nm]	FWHM [nm^{-1}]	CCL [nm]
w/o	4.08/1.51	1.39	6.07	17.8/0.353	3.39	2.50
DOB	4.10/1.51	1.37	6.16	17.8/0.353	3.16	2.68
OIB	4.06/1.55	1.07	7.89	17.9/0.351	3.02	2.81
DIB	3.36/1.87<br>4.30/1.46	0.786<br>0.807	10.7<br>10.4	18.0/0.349	2.65	3.20
SIB	3.39/1.85<br>4.48/1.41	0.326<br>0.539	25.9<br>15.7	18.0/0.349	2.05	4.14
DSB	4.05/1.55	0.204	41.4	17.5/0.359	1.11	7.65

	4.68/1.34	0.274	32.5	18.9/0.332	0.98	8.67

Table S3. Photovoltaic parameters of PBBTz-Cl:PY-IT based all-PSCs without and with SIB additive.

SIB	V_{oc} [V]	J_{sc} [mA cm^{-2}]	J_{EQE}[b)] [mA cm^{-2}]	FF [%]	PCE [%]
w/o	0.948	23.82	23.17	69.23	15.63
	0.951±0.002[a)]	23.72±0.31		67.91±0.75	15.32±0.27
50 wt%	0.946	24.87	24.66	74.23	17.47
	0.952±0.005	24.37±0.51		74.12±0.60	17.19±0.42

[a)]The averaged values with standard deviations were calculated from 10 individual devices; [b)]The integrated current densities were calculated from the EQE curves.

Table S4. Photovoltaic parameters of PM6:PBTz-F:L8-BO based OSCs with different weight ratios of PM6:PBTz-F.

PM6:PBTz-F	V_{oc} [V]	J_{sc} [mA cm^{-2}]	J_{EQE}[b)] [mA cm^{-2}]	FF [%]	PCE [%]
1:0	0.898	26.30	25.46	79.92	18.87
	0.896±0.005[a]	26.27±0.19		78.96±0.91	18.58±0.24
0.8:0.2	0.907	27.10	25.90	80.06	19.68
	0.906±0.005	26.93±0.13		78.96±0.77	19.27±0.23
0.7:0.3	0.909	26.91	25.82	79.22	19.37
	0.901±0.007	26.87±0.14		78.69±0.87	19.05±0.31
0:1	0.905	26.57	25.55	78.23	18.81
	0.908±0.005	26.31±0.27		77.56±0.59	18.53±0.29

[a)]The averaged values with standard deviations were calculated from 10 individual devices; [b)]The integrated current densities were calculated from the EQE curves.

References

[1] B. Pang, C. Liao, X. Xu, L. Yu, R. Li, Q. Peng, *Adv. Mater.* **2023**, *35*, 2300631.
